# Mandibular Asymmetry Index and Dental Occlusion in Patients with Temporomandibular Disorders Treated with Occlusal Splint

**DOI:** 10.3390/dj13040176

**Published:** 2025-04-20

**Authors:** Sergio Paduano, Raffaella Grimaldi, Ludovica Nucci, Mario Fordellone, Rossana P. Rotolo, Vincenzo Grassia, Fabrizia d’Apuzzo

**Affiliations:** 1 Department of Health Sciences, University Magna Graecia of Catanzaro, Viale Europa, 88100 Catanzaro, Italy; paduano@unicz.it; 2 Department of Surgical Sciences, University of Cagliari, Via Università 40, 09124 Cagliari, Italy; grimaldi.raff@gmail.com; 3 Department of Mental and Physical Health and Preventive Medicine, University of Campania Luigi Vanvitelli, Largo Madonna delle Grazie 1, 80138 Napoli, Italy; ludovica.nucci@unicampania.it; 4 Medical Statistics Unit, University of Campania Luigi Vanvitelli, Via Luciano Armanni 5, 80138 Naples, Italy; mario.fordellone@unicampania.it; 5 Multidisciplinary Department of Medical-Surgical and Dental Specialties, University of Campania Luigi Vanvitelli, Via Luigi De Crecchio 6, 80138 Napoli, Italy; vincenzo.grassia@unicampania.it (V.G.); fabrizia.dapuzzo@unicampania.it (F.d.)

**Keywords:** temporomandibular disorders, occlusal splint, asymmetry, mandibular condyle, malocclusion

## Abstract

**Objectives:** To evaluate any changes in condylar and mandibular ramus height and dental malocclusion in adult patients with temporomandibular disorders (TMDs) diagnosed with DC/TMD criteria after treatment with an upper occlusal splint. **Methods:** This retrospective observational study included 48 adult patients with TMDs treated with an occlusal splint in the upper arch for about 12 months. For each patient, panoramic dental X-rays were analyzed using the Habets method to calculate the asymmetry index between the condyles and mandibular branches before and after treatment. The digital dental models were also studied at T0 and T1 to define the occlusal sagittal molar relationship and the posterior dental crossbite. The statistical analysis was performed using the Shapiro–Wilk normality tests, Student *t*-tests, or Wilcoxon tests using the R studio software (released version 4.3.3). **Results:** Condylar height showed a statistically significant difference (*p* = 0.022) and reduced condylar asymmetry between T0 and T1. The measurement of the condylar branch showed a statistical significance (*p* = 0.037), revealing an improvement of the mandibular symmetry in the vertical direction after treatment. Moreover, at T0, posterior dental crossbite was found in 37.5% of patients, specifically, bilateral in 12.5%, while unilateral crossbite in 22.9% on the right and 2.1% on the left side, and Class I malocclusion was found in the main part of the sample (72.9%). **Conclusions**: Patients with TMDs diagnosed according to DC/TMD and treated with an upper stabilization occlusal splint in the case of symptoms of masticatory muscle dysfunction showed a symmetrization in the mandibular ramus and condyle pre- and post-treatment (T0-T1). At the same time, no clinical differences were found in the occlusal characteristics.

## 1. Introduction

Temporomandibular disorders (TMDs) involve temporomandibular joint (TMJ), masticatory muscles, and/or dental structures. Many factors, including personality traits and psychological, occlusal, traumatic, emotional, and parafunctional factors [1,2], can cause TMDs.

Orofacial pain in the periauricular area is usual and associated with difficulty chewing and swallowing, tension headache, muscle or joint tenderness on palpation, limitation of mandibular movements, joint sounds, tinnitus, vertigo, or ear fullness [3,4,5].

The incidence of TMDs in the world is 34% [6]. It varies by geographical area, and indeed the prevalence is significantly greater in South America (47%) compared to Asia (33%) and Europe (29%). TMDs are one of the most common sources of pain in the orofacial region [7,8].

TMDs are a serious public health problem, as they are one of the main sources of chronic orofacial pain that interferes with quality of life. These conditions are also frequently associated with headaches, cervical spine dysfunction, and modified head and cervical posture [9].

Nowadays, the diagnostic criteria for TMDs (DC/TMD) are most followed worldwide [10]. They are divided into two sections: Axis I comprises the clinical examination and categorizes TMDs into three groups which are group I—muscular disorders (Ia myofascial pain; Ib myofascial pain with mouth opening limitation), group II—disc displacement (IIa disc displacement with reduction, IIb disc displacement without reduction with limited opening, IIc disc displacement without reduction and without limited opening), and group III—arthralgia (IIIa), arthritis (IIIb), and arthrosis (IIIc). Moreover, Axis II of the DC/TMD evaluates the pain related to the disability, calculating the treatment effect and the chronic pain [10].

Treating chronic pain of the orofacial muscles is complicated in clinical practice, based on the degree of muscle impairment and the gravity of symptoms. Non-invasive and reversible treatment of TMDs, including exercise, physiotherapy, pharmacological interventions (such as nonsteroidal anti-inflammatory drugs, muscle relaxants, or narcotic analgesics), acupuncture, intra-articular injections with anesthetics or corticosteroids, psychological, surgery, and splint therapy often removes the pain. Occlusal splints are one of the typical treatments of TMDs, and numerous research confirmed their effectiveness on masticatory muscles [11,12,13,14,15,16,17].

Various designs of these appliances are available, i.e., stabilization and anterior repositioning splints, but the most used and documented, is the stabilization one [18,19].

The therapeutic effect is provided by various peripheral, central, and behavioral changes. Effects of occlusal splints include reduced muscle activity, improved occlusal stability, increased vertical size, cognitive alterations, and placebo effect [20].

The main indications for using splints for the treatment of TMDs are tension-type headaches, articular disc disorders, and the prevention of excessive wear of the teeth in patients with parafunctional habits, such as bruxism [21]. Muscle contraction seems to be a significant aspect of TMDs in which anxiety, stress, parafunction, and arthralgia are the main etiological factors.

The articular surface may be overloaded due to muscle hyperactivity, leading to the progress of osteoarthritis with the thickening of soft tissues, specifically the undifferentiated mesenchymal cell layer. There is an increase in condylar asymmetry. This process can continue until the ability to adapt is at its limit. This is where TMJ problems come in [1,22,23].

Condylar asymmetry (CA) between right and left mandibular condyles may be useful for diagnostic and monitoring purposes in patients with TMDs [24]. Habets et al. examined CA and observed higher asymmetry in the vertical condylar height of the TMDs patients compared with controls [25,26]. Furthermore, Bezuur et al. assessed CA in TMDs patients and found most of them with a vertical CA [27]. Habets et al. established a system to calculate asymmetry between the condyles, comparing the vertical height of the right and left mandibular condyles. In that technique, the CA index (CAI) was assessed using orthopantomographic images (OPGs) and was defined as the ratio of the difference between the right and left condylar heights compared with their total [26]. The CAI has been used to validate clinical tests for diagnostic purposes in patients with temporomandibular disorders [6]. Other assessment systems for OPGs were explained by other authors, such as Lemos, Levandoski, and Kjellberg [1].

Only a few studies have applied the Habets method to evaluate condylar asymmetry in TMDs patients. Based on background, the hypothesis seems to be that patients with TMDs have a higher possibility of showing facial and skeletal asymmetry, while a counter hypothesis is that there is no implication on the mandibular condyles.

Thus, to better focus on this unresolved issue, the present study aimed to measure the mandibular ramus and condyle heights on the right and left sides before and after treatment with an upper occlusal splint to check the eventual vertical asymmetry in adults with TMDs. The secondary objectives were to assess digital dental models before and after treatment to assess any changes in dental malocclusion (molar relationship, and posterior dental crossbite) after the use of the upper splint.

## 2. Methods

The protocol of this observational retrospective cohort study was previously approved by the Ethics Committee (Prot n. 0009122/i, Approved on 24 March 2023) of the University of Campania *Luigi Vanvitelli*, Naples (Italy) before starting the data collection. All subjects had signed informed consent for the use of personal information. Records of patients were collected from March 2023 to June 2023 from the archives of the Orthodontic Program of the same University.

The inclusion criteria were as follows: (1) patients aged between 18 and 70 years old; (2) good general health; (3) diagnosis of TMDs (specifically excessive activity and pain of the masticatory muscles) using clinical and psychosocial assessments following the DC/TMD criteria; (4) treatment with an upper occlusal stabilization acrylic splint for about one year; (5) good quality of OPGs and digital dental models before and after treatment.

Specifically, the OPGs should have no distortions with clear visualization of mandibular condyles and rami to allow correct measurements on them.

Patients were excluded if they continuously used drugs that could have affected balance, visual impairment, neurological problems, labyrinthitis as well as analgesic, anti-inflammatory, or muscle relaxant drugs, or patients with removable dental prostheses, congenital or acquired facial anomalies (i.e., condylar hyperplasia), a history of facial fractures, previous maxillofacial surgery.

OPGs and dental models are routinely requested by each subject undergoing a dental visit because they provide initial information on the patient’s dentition and temporomandibular joints to carry out an initial diagnostic classification, and then to proceed to further investigations. No additional radiological imaging procedures were necessary for this study, following the ALARA concept of radioprotection.

The DC/TMD criteria are a tool to make a diagnosis of different types of TMDs and its algorithms have been demonstrated to be reliable and validated worldwide for the most common pain-related TMDs and intra-articular disorders.

The occlusal stabilization splints were used to provide orthopedic stability to the joints. This stabilization type of splint is made of hard acrylic resin and covers all teeth on either the maxillary or mandibular arch.

It is registered at the chair by the specialist to have all teeth in occlusion when the patient closes the jaw in a musculoskeletal stable position. The therapeutic rationale for using these splints was to provide temporary and ideal occlusion, reduce muscle function, and protect teeth from clenching activities. A standard model of the bite was then adapted at the chair by the specialist, considering the occlusion and parafunctions of each subject.

Regular follow-ups every 4 weeks by specialists in orofacial pain were scheduled to register the splint to the chairside with an articulating paper of 200 microns of thickness used to ensure symmetrical and similar occlusal contacts with the same intensity in closing the mouth in every subject.

Patients were advised to wear these appliances on the upper arch mainly at night for a maximum time of 12 h per day. Clinical steps were performed and monitored by the same two experienced orthodontists.

For each patient, the following data before and after treatment were evaluated:

condylar and ramus height on OPG with the calculation of the symmetry index and posterior dental crossbite and molar relationships to evaluate Angle malocclusion on dental models.

**Figure 1:**
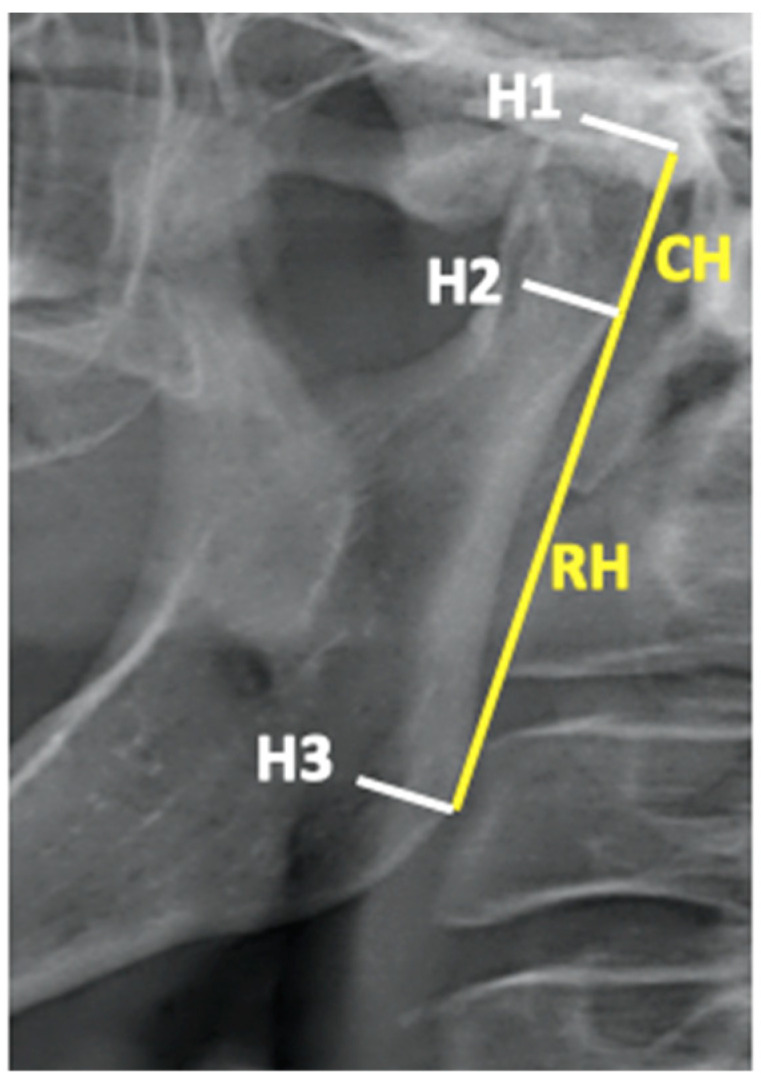
Habets’ method [25,26].

The method described by Habets to calculate the asymmetries of the condyles and rami compared the vertical heights of the right and left condyles and rami on OPGs (Figure 1).

The operator outlined the mandible, embracing the condyles, on acetate paper. On each side of the outline of the mandible, a line tangent to the most lateral point of the ramus and the most lateral point of the condyle (line V) was drawn, followed by three perpendicular lines: one tangent to the highest point of the condyle (line H1); one intersecting line V at the most lateral point of the condyle (line H2); and one intersecting line V at the most lateral point of the ramus (line H3). Subsequently, the operator measured the distances H1/H2 and H2/H3 on each side with digital calipers. The operator that performed the tracings was blind, i.e., unaware of the patient’s allocation to the experimental groups. Therefore, the height of the OPG image of the condyle corresponded to the distance H1/H2 and the height of the OPG image of the ramus corresponded to the distance H2/H3. To assess the asymmetry between the right (R) and left (L) condyles and rami (asymmetry index), the formula |(R − L)/(R + L)| × 100 was used. An asymmetry index ≤6% indicated that the left and right structures were to be considered symmetrical, whereas a result >6% was indicative of asymmetry. The 6% threshold accounts for slight rotations in patient positioning during the exposure of the OPG, resulting in asymmetrical enlargement of the structures of interest [25,26,27].

The transversal dental malocclusion was checked on the digital dental model and measured at the molar. The sagittal dental relationship following Angle’s classification of malocclusions was based on the relative position of the permanent maxillary first molar. Specifically, a normal molar relationship presents the mesiobuccal cusp of the maxillary first molar occluding in the buccal groove of the mandibular first molar [28].

### Data Analysis

Continuous variables were reported as either the means and standard deviation or median and interquartile ranges (IQRs) according to their distribution, as assessed by the Shapiro–Wilk normality test. Categorical variables were reported as percentages. Differences in characteristics of patients before and after the treatment (i.e., at T0 and T1) were tested by *t*-test for paired samples or Wilcoxon sign-rank test (according to their distribution) and McNemar’s test for continuous and categorical variables, respectively. The Shapiro–Wilk test was statistically significant; therefore, all distributions are not normal.

**Figure 2:**
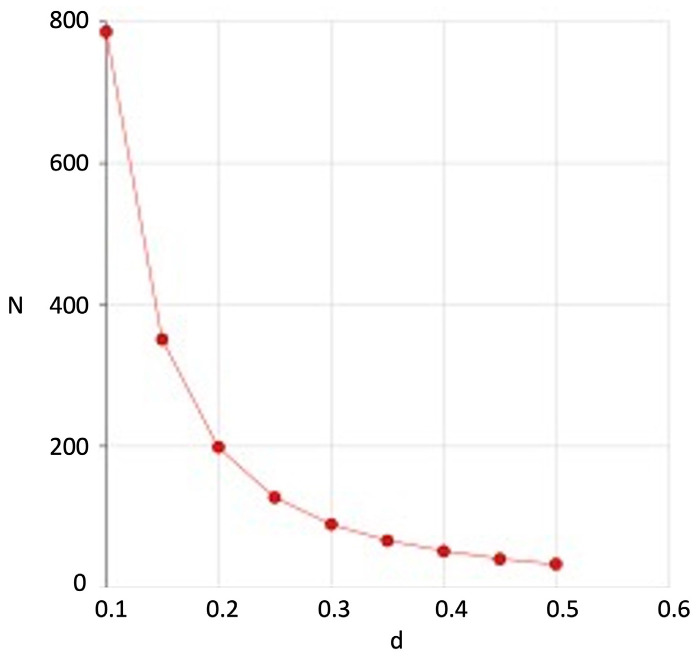
Sample size number for the effect size based on the power of 80% and a significance level (alpha) of 0.05 using a two-sided Wilcoxon signed-rank test for paired data.

A preliminary power analysis was performed to estimate the maximum sample size necessary to detect an effect size (i.e., the standardized mean difference between Condyle and Ramus symmetry index at T0 and T1) equal to 0.40. With a sample size of a maximum of 48 pairs of data, the analysis achieves a power of 80.78% in rejecting the null hypothesis that the effect size is equal to zero. This result is based on a significance level (alpha) of 0.05 and utilizes a two-sided Wilcoxon signed-rank test for paired data (Figure 2). For interpretive purposes, we considered an effect size of 0.20 as small, 0.50 as medium, and 0.80 as large [29]. All the statistical values equal to or smaller than 0.05 were considered statistically significant. The analysis was conducted using the R statistical package (released version 4.3.3).

## 3. Results

Starting from 72 selected subjects, 11 were excluded for missing dental OPGs, 7 were excluded for missing dental models at T1, and 6 were excluded because they had undergone surgical and/or prosthetic interventions during the upper splint treatment.

**Figure 3:**
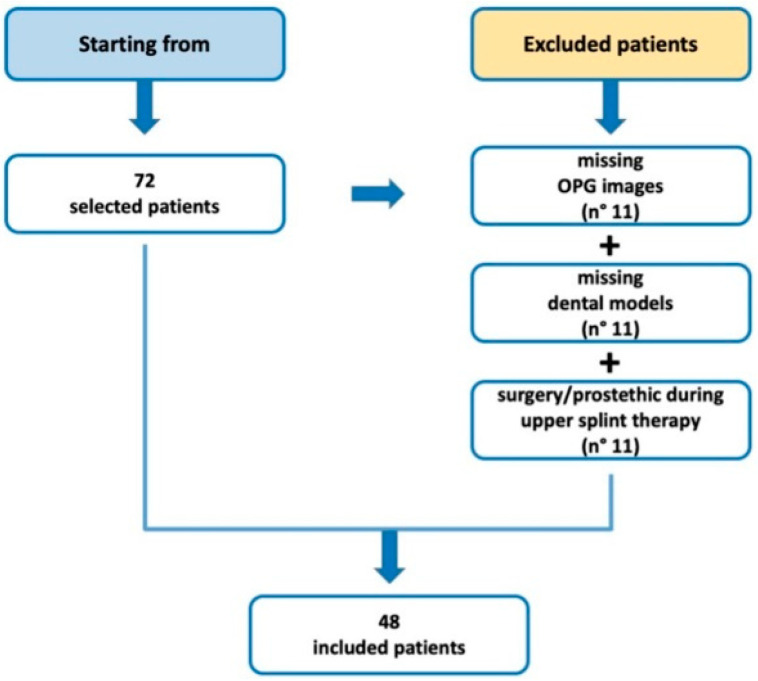
Flowchart of included patients in the study group considering the selection criteria.

Thus, a total of 48 patients were included in the final analysis after the collection of the data before and after the treatment (Figure 3).

The study included 48 patients (41 females and 7 males) with a mean age of 26.92 ± 19.82 years.

Table 1 showed the occlusal characteristics of patients before the splint therapy. At baseline T0 the sample presented an Angle Class II relationship in 10 subjects (20.8%), Angle Class III in 3 subjects (6.2%), and Angle Class I in 35 patients (72.9%).

Moreover, 6 patients manifested a bilateral crossbite (12.5%), 11 patients had a crossbite on the right side (22.9%), 1 patient on the left side (2.1%), and 30 patients presented no crossbite (62.5%).

The duration of the upper splint treatment (T0-T1) was, on average, 21.22 months.

No occlusal changes were found after splint therapy.

Statistical significance was found by analyzing condylar symmetry values before and after treatment with upper splint therapy evaluated on the orthopantomographic examination according to the Habets method. Specifically, condylar height showed a statistically significant difference with *p* = 0.022, showing a reduction in condylar asymmetry post-treatment.

**Figure 4:**
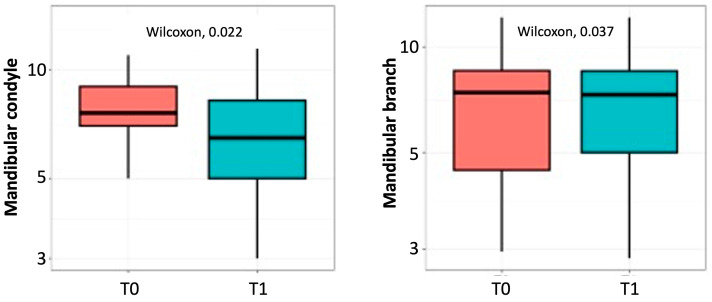
Condylar and Ramus symmetry index (Habets method) before (T0) and after (T1) treatment.

The measurement of the ramus height equally showed statistical significance with *p* = 0.037, showing an improvement in mandibular symmetry in the vertical direction after the treatment in adult subjects (Figure 4).

## 4. Discussion

As reported in the literature, the association between vertical mandibular asymmetry and TMDs remains controversial [1,23,30].

Only a few studies have applied the Habets method to evaluate condylar asymmetry in TMD patients. Specifically, Tortarolo et al. used it to assess the mandibular asymmetry in children (mean age ± SD = 8.0 ± 1.3 years) with unilateral posterior crossbite pre-treatment. The results underlined a significantly augmented asymmetry of condyles (mean ± SD = 10.7% ± 9, *p* < 0.001), but not of rami [31].

Buranastidporn et al. used the posteroanterior cephalograms of adults (mean age of 23.9 years) before orthodontic treatment to examine the inclination of the frontal occlusal and mandibular planes to determine vertical asymmetry. They concluded that the degree of vertical asymmetry was significantly related to the presence of TMD symptoms and recommended a vertical assessment when initiating a treatment plan to correct the occlusal functions [24].

Yanez-Vico et al. found that the mandibular asymmetry calculated on 3D-computed tomography was a more common characteristic in patients with TMDs aged between 25 and 42 years than the control group (*p* < 0.05) [32].

Karic et al. examined the relationship between the condylar asymmetry and the temporomandibular opening index (the end feel distance divided by active and passive mouth opening) in myogenic TMDs and a control group without TMDs. A high degree of asymmetry (*p* = 0.001) and a significant positive correlation between the condylar asymmetry and the temporomandibular opening index only in the myogenous TMDs group (r = 0.84; *p* = 0.001) was found [33].

Other authors suggested a possible association between young adults (mean age 16.8 years) with facial and/or condylar asymmetry and displacement of the articular disc [34].

Conversely, other research has reached different results. Säglam et al. did not find statistically significant results to suggest an association between the presence of condylar asymmetry and TMDs, finding less asymmetry of the mandibular condyle than other authors [1,35].

Our results showed a significant improvement in mandibular symmetry after treatment with the upper occlusal splint on dental panoramic X-rays before and after treatment. To avoid any selection or performance biases, the OPGs were accurately evaluated to retrospectively include in our sample only good-quality X-rays. No image distortions due to positioning errors were found and nor were any issues in this selection due to the special attention to the visualization of the mandibular condyles posed by the specialist previously requiring this diagnostic examination in an adult population.

Therefore, there is no certain evidence to determine the possible effects of the amount of condylar asymmetry on the clinical frame of patients affected by some TMDs.

To note, any restrictions on age were not applied in our study because the objective was to evaluate the condylar and mandibular asymmetry in a wide sample of treated adult patients with no more vertical mandibular growth being the minimum age of the sample equal to 18 years. Also, patients with congenital or acquired craniofacial anomalies, such as condylar hyperplasia with possible elements of continued condylar growth, were excluded from the beginning of our assessment [36].

The secondary objective of our investigation was to assess the presence of dental malocclusion (molar and cuspid relationship and posterior crossbite) before and after treatment in our sample with TMDs. The results at T0 showed the presence of Angle Class I in most patients followed by Class II and Class III relationships, with no consistent changes at T1. Prevalences of dental malocclusion in our sample were in line with the mean percentages reported in a Caucasian population [37]. However, no other studies investigated the occlusion before and after treatment with the occlusal splint in patients with TMDs. These outcomes are congruous with the rationale of using stabilization splints to non-invasively treat the patients affected by different types of joint disorders, which should only correct vertical parameters but not change the transversal and sagittal dental occlusion after one year of wearing the splint mainly at night [38,39].

An accurate statistical analysis using the effect size calculation in clinical studies underlines the quality of research in this field of TMDs, which still needs to be deeply investigated in our patients suffering from orofacial pain [40].

The limitations of the study were the retrospective nature and the use of a single type of treatment with other types of treatment (i.e., jaw exercise, non-steroidal anti-inflammatory drugs, arthrocentesis, transcutaneous electrical nerve stimulation, etc.) as suggested by current guidelines [41].

In addition, some more insights into the efficacy of occlusal splints on mandibular asymmetry would have been discussed in a comparison with a control group. However, it was not allowed in a hospital setting to postpone the treatment of these patients with TMDs and mandibular asymmetry due to clinical/ethical requirements.

Thus, future purposes of this research topic could be to integrate the mandibular evaluation also using lateral cephalograms with the cephalometric analysis of the skeletal parameters relating to mandibular divergence of the anterior and posterior facial heights in the vertical direction, and incisor inclinations after splint treatment and/or other types of treatment [42], through multicentric prospective studies in a different sample of patients.

Further research is highly welcome to compare the measurements of the Habets method on panoramic X-rays to those carried out with a three-dimensional evaluation as the cone beam computed tomography (CBCT) with higher diagnostic accuracy.

Moreover, to these static morphological analyses, the integration with magnetic resonance imaging should provide the researchers in this field with some interesting information on the ‘biotensensegrity’ [43]. This is a recent concept that describes how the mandible is suspended in a network of tensioned ligaments, muscles, and fascia, which may help to explain the possible reasons for the acquired condylar asymmetry to respond to changing demands during mandibular growth in younger age.

Finally, within the study’s limitations, we think that these outcomes could be a start for considering the OPG as the first assessment on a wider scale of the mandibular condyles during the first dental visits for each patient to evaluate the possible correlation with TMD eventually more deeply through the request of further imaging evaluations and other consultations with experts in different dental and medical specialties.

## 5. Conclusions

Patients with TMDs diagnosed according to DC/TMD and treated with an upper stabilization occlusal splint in the case of symptoms of masticatory muscle dysfunction showed an improvement in the symmetry of mandibular ramus and condyle pre- and post-treatment (T0-T1). At the same time, no clinical differences were found in the occlusal characteristics.

## Figures and Tables

**Table 1:** Occlusal characteristics (molar and cuspid relationship, presence of crossbite) at baseline (T0).

Sample	N = 48 ^1^
***Age, years***	**26.92 (19.82)**
***Crossbite***	
*Bilateral*	6.0 (12.5%)
*On the Right Side*	11.0 (22.9%)
*On the Left Side*	1.0 (2.1%)
*None*	30.0 (62.5%)
***Angle Class Relationship***	
*Class I*	35.0 (72.9%)
*Class II*	10.0 (20.8%)
*Class III*	3.0 (6.2%)
***Time Tx, months***	**21.22 (9.46)**

^1^ Median (IQR) or Frequency (%).

## Data Availability

The original contributions presented in this study are included in the article. Further inquiries can be directed to the corresponding author.
